# Supplementary material for: Primary Amine Functionalized Carbon Dots for Dead and Alive Bacterial Imaging
Source: Nanomaterials (Basel). 2023 Jan 21;13(3):437. doi: 10.3390/nano13030437 (PMC9920602; doi:10.3390/nano13030437)
Supplement: Supplementary file 1 [file nanomaterials-13-00437-s001.zip › nanomaterials-2150557-supplementary.pdf]

nanomaterials

Supplementary Materials

Primary Amine Functionalized Carbon Dots for Dead and Alive Bacterial Imaging

Yuting Liu [1], Di Zhong [2], Lei Yu [1], Yanfeng Shi [1] and Yuanhong Xu [1,*]

1 Institute of Biomedical Engineering, College of Life Science, Basic Medical College, Qingdao University, Qingdao 266071, China
2 Department of Genetics and Cell Biology, Basic Medical College, Qingdao University, No. 308 Ningxia Road, Qingdao 266000, China
* Correspondence: yhxu@qdu.edu.cn

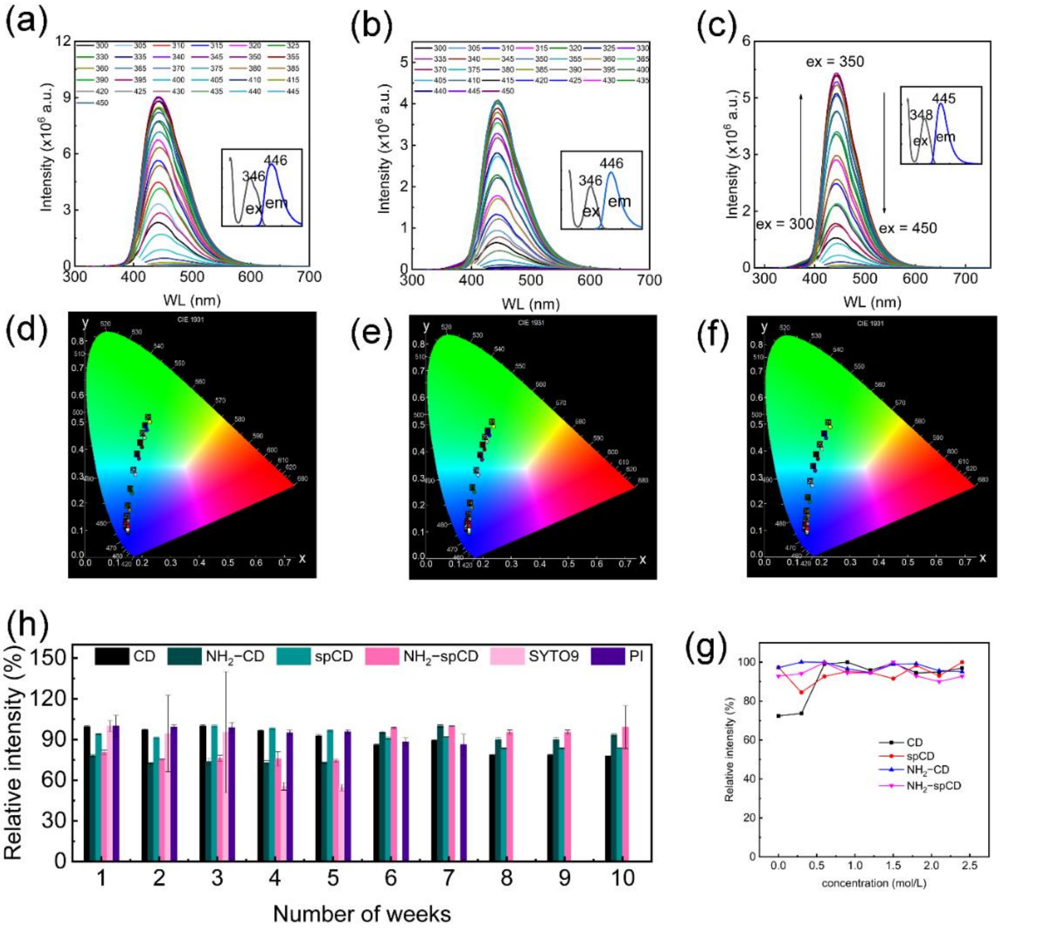

Figure S1. (**a**) FL emission map and ex-em spectrum of CDs, (**b**) NH_2–CDs and (**c**) NH_2–spCDs; (**d**) CIE 1931 chromaticity chart of CDs, (**e**) NH_2–CDs and (**f**) NH2–spCDs at excitation of 300~450 nm (step = 5 nm); (**h**) Relative intensity changes of CDs, spCDs, NH_2–CDs, NH_2–spCDs and two dyes (SYTO9 and PI) for a long-term placement; (**g**) Relative fluorescent intensity of CDs, spCDs, NH_2–CDs, and NH_2–spCDs under different concentration of NaCl solutions.

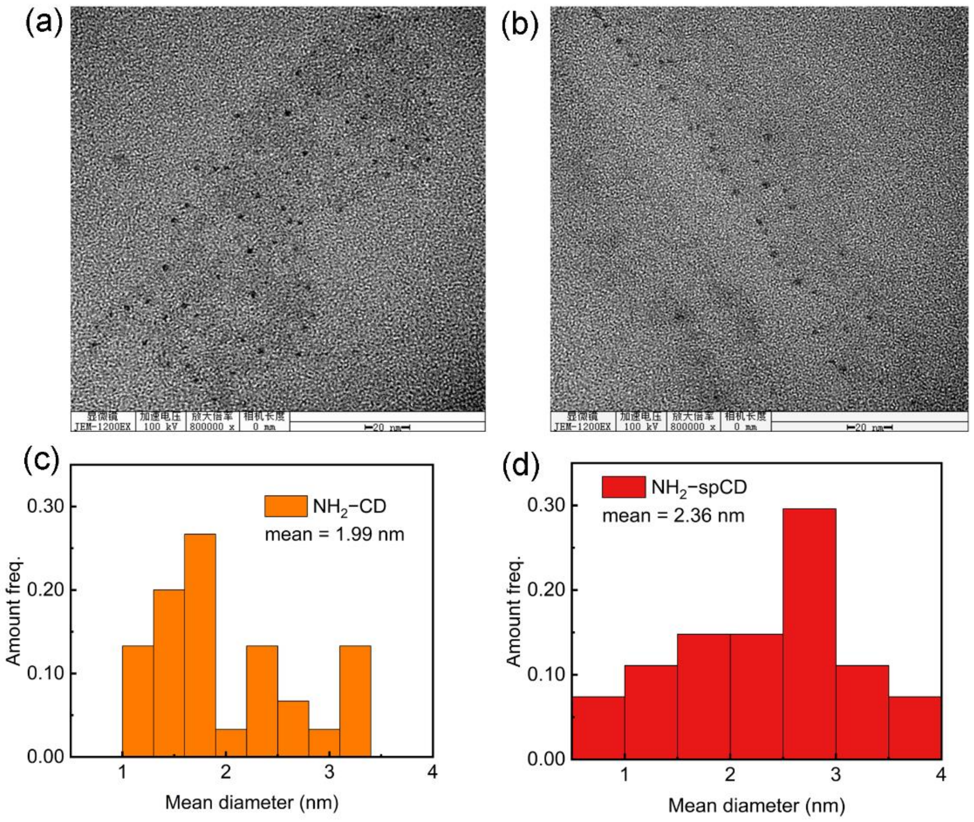

Figure S2. TEM of (**a**) NH$_2$–CDs and (**b**) NH$_2$–spCDs; size distribution of (**c**) NH$_2$–CDs and (**d**) NH$_2$–spCDs.

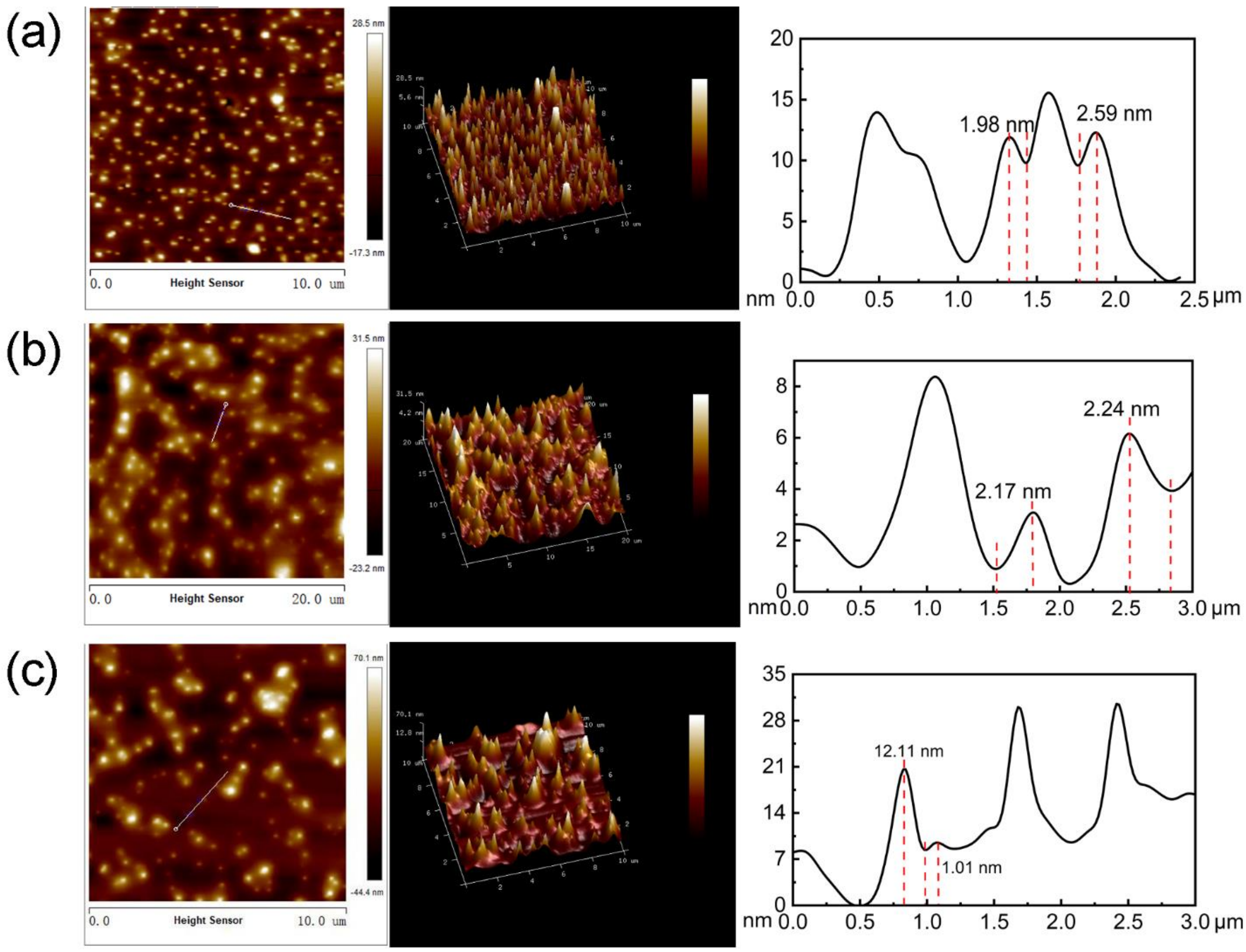

Figure S3. AFM of (**a**) CDs, (**b**) spCDs, (**c**) NH_2–CDs (left: 2D images; middle: 3D images corresponding to the 2D images; right: measurements of height on the left images).

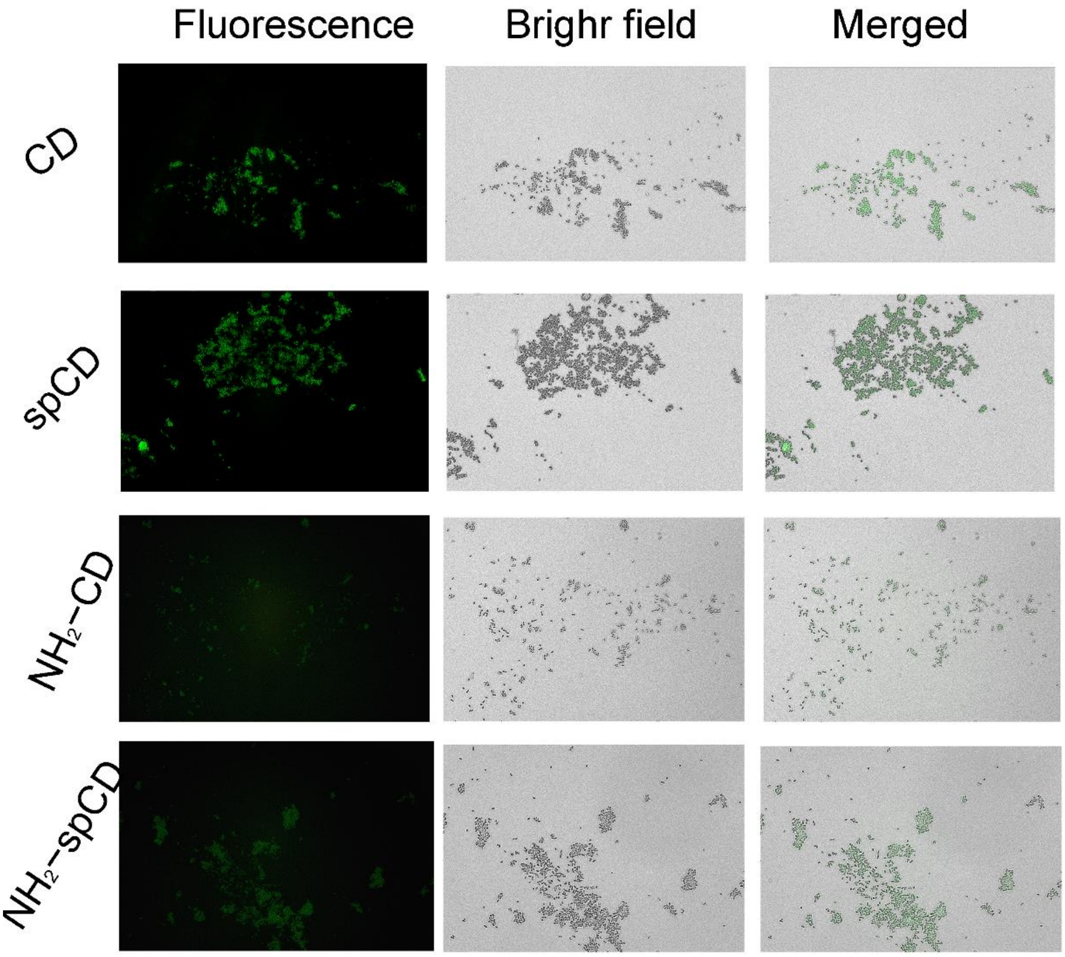

Figure S4. Imaging of E.coli by CD, spCD, NH_2–CD, and NH_2–spCD (bacterial was treated with 3% benzalkonium bromide).

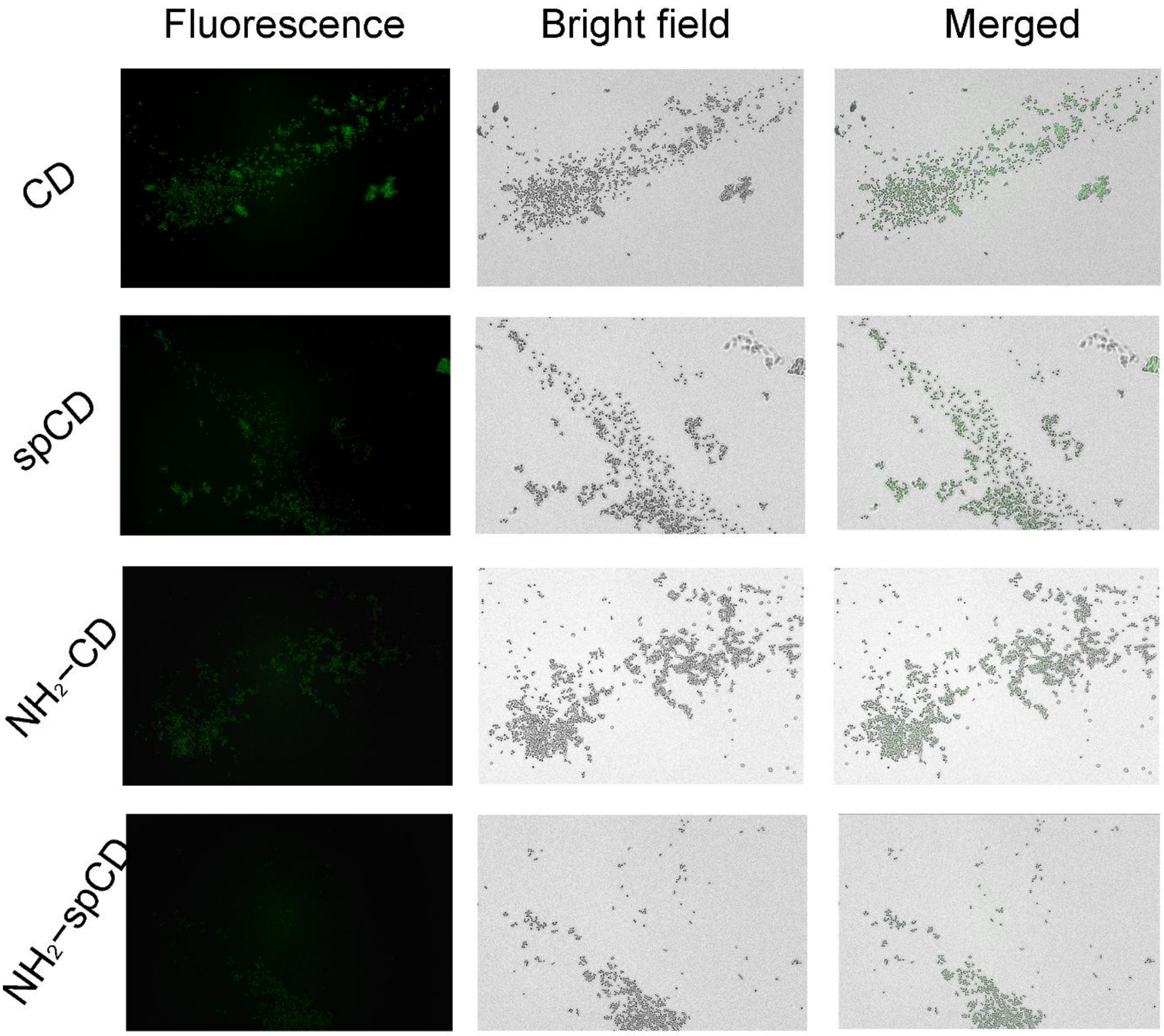

Figure S5. Imaging of *S.aureus* by CDs, spCDs, NH_2–CDs, and NH_2–spCDs (bacterial was treated with 3% benzalkonium bromide).

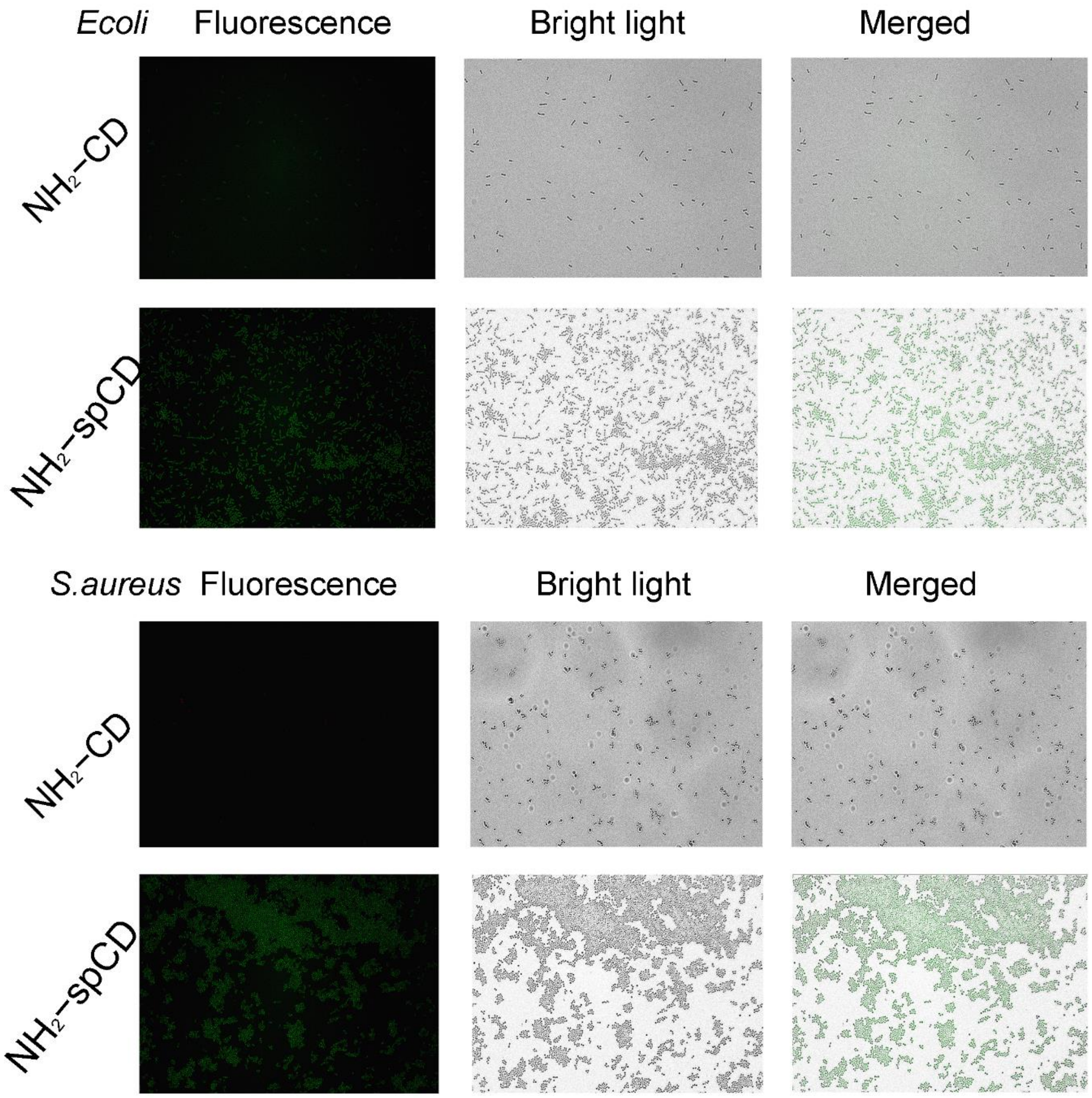

Figure S6. Imaging of dead *E.coli* and *S.aureus* by NH_2–CDs and NH_2–spCDs (bacterial was treated with 60 °C water bath for 1 hour).

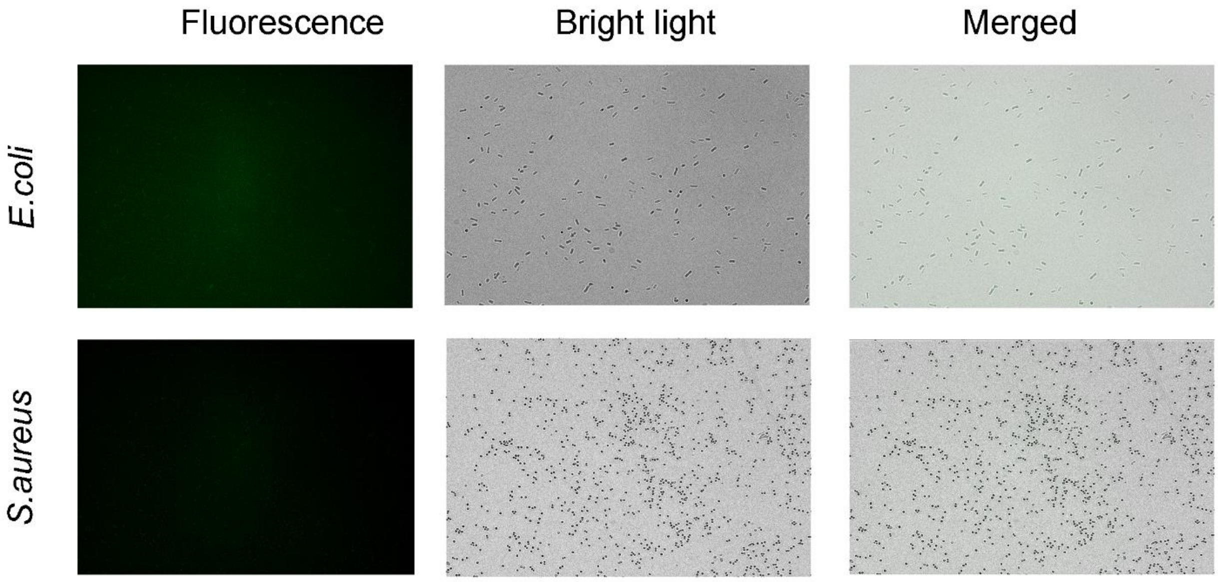

Figure S7. Imaging of alive *E.coli* and *S.aureus* by NH_2–CDs.
